# Identifying Protein Function—A Call for Community Action

**DOI:** 10.1371/journal.pbio.0020042

**Published:** 2004-03-16

**Authors:** Richard J Roberts

## Abstract

Functional information is lacking for many of the hypothetical proteins encoded within sequenced genomes. Richard Roberts proposes that a community-based approach might offer an effecient way to fill the function gap

During the last few years, we have seen enormous strides in our abilities to sequence genomes, and the information that has poured out of these sequences is quite astonishing. With more than 150 complete genome sequences now available and many laboratories rushing into microarray analysis, proteomic initiatives, and even systems biology, it seems an appropriate time to consider not just the opportunities those sequences present, but also their shortcomings. By far the most serious problem is the quality and degree of completeness of the annotation of those genomes. Most troublesome are the large numbers of open reading frames that have been identified by computer programs, but remain labeled as a “conserved hypothetical protein” when they occur in more than one genome or simply a “hypothetical protein” when they appear unique to the genome in question. Between them, these two categories of annotated open reading frames often represent more than half of the potential protein-coding regions of a genome.

These annotations highlight just one portion of our ignorance about the information content of genomes and our lack of fundamental knowledge about the function of so many of the building blocks of cells. Unless we rectify this situation, it is likely to undermine many of the other “-omic” efforts currently underway. Here I advocate a rather straightforward approach to address this problem—focused initially on the bacterial genomes. In contrast to the numerous proposals for big science initiatives to understand the fundamental workings of biological organisms, I propose a small science, relatively low-tech approach that could have a dramatic pay off. A relatively small investment could yield a massive amount of information that would greatly enhance our current efforts to use genomic approaches to study life.

## Initial Proposal

The initial proposal is directed at deciphering the role of the “hypothetical proteins” encoded in the microbial genomes and would involve a community-wide approach to determine the function of these hypotheticals based on solid, old-fashioned biochemistry. The essence of the idea is to undertake an interdisciplinary effort that couples our current bioinformatics capabilities to predict protein function with a directed exploration by experimental laboratories to test those predictions. I would encourage a consortium of bioinformaticians to produce a list of all of the conserved hypothetical proteins that are found in multiple genomes, to carry out the best possible bioinformatics analysis, and then to offer those proteins to the biochemical community as potential targets for research into their function. To energize laboratories with appropriate expertise to participate in this community-wide effort, I suggest that a special program be set up by one or more of the funding agencies so that laboratories undertaking the investigation of any particular protein receive a small grant upfront as a supplement to an existing grant. Upon completion of the project and the identification of the function, they would receive a further supplement to that grant as a reward. In this way, one might hope to rally some of the best biochemical talent and apply it to this problem of determining function for a wide range of new proteins. The cost of such an operation could be quite minimal, and the bureaucracy and review process could be equally simple. Here is a case where a modest infusion of funds could greatly enhance our ability to annotate both existing and new genome sequences and ensure that our current investments in genomic sequences yield the richest biological harvest possible. There are two key steps in the proposed plan.

## Key Steps

The first step is to encourage some bioinformaticians with appropriate expertise in the functional annotation of genomes to form a consortium and undertake the assembly of a list of prime targets for which an experimental demonstration of function would be most valuable. Three general classes of such genes come to mind: (1) The conserved hypothetical genes. These belong to the set of genes that have orthologs in many other genomes, but for which no function has been experimentally determined in any case. A recent success among such genes is illustrated in Box 1. (2) The hypothetical genes. These form the set of genes that are predicted to be protein coding, but that lack similar genes in any other organism in GenBank. They, too, have no assigned function. (3) The misannotated genes. These genes are ones for which a function has been assigned, but for which there is a good reason to believe the annotation is incorrect.

These sets of targets would be combined and arranged into a prioritized list in which each was accompanied by the best assessment of potential function. The priorities would be based on which genes were most likely to prove broadly informative. For instance, a conserved hypothetical gene that occurred in most genomes would be of higher priority than one that had only two orthologs. The list would be on a public Web site where these targets and the predicted functions could be examined and modified by alternative or additional predictions from other groups to guide future experimentation. As function was derived, that information could be presented and the target removed from the main list.

The second step would be to invite experimentalists to peruse the list and find those potential genes whose protein products might lie within their realm of expertise so that they could use their experimental knowledge and reagents to quickly test for function. Initially, I would advocate allowing laboratory teams to pick and choose among the list and sign up to study just one of these open reading frames. I would recommend allowing one laboratory per open reading frame in the initial stages. A laboratory wishing to sign up would generate a short document highlighting why its expertise might be suitable for a particular protein. A one-page proposal should suffice, with no experimental plan demanded. At this point, a small panel could choose among competing efforts and the laboratory chosen would be given a small grant and up to six months to carry out its analysis. If it was successful in delineating the function of their target protein, a paper would be written and submitted for peer review. If the paper was accepted for publication, then an additional sum would be allocated as a supplement to the laboratory's existing grant. If, after six months, a laboratory had not managed to delineate the function, it would submit a short report describing the approaches that have been tried, with the results of its analyses. This would be posted on the public Web site and that target would then become open for analysis by other laboratories, under the same conditions as before.

While the initial list of target genes should probably be based on a well-studied and experimentally tractable organism such as Escherichia coli, I would not demand that the biochemical experiments be done on the E. coli gene. Any of the orthologs would do, so long as the similarity was sufficiently strong to give high expectations that function would be conserved. In fact, for a laboratory that happened to be already working on one of the homologs, this program might provide an added bonus and greatly speed its work. I would also encourage both biochemical and genetic approaches, since one can never be certain when one method might be better than another. The list would, of course, also include conserved genes not found in E. coli, but commonly distributed in other genomes. In particular, I would make a pitch for including all genes in Mycoplasma genitalium, which, as the free-living organism with the fewest genes, might be the most suitable as a model system for in-depth understanding of its biology.

## The Importance of Community

This proposal for experimental attack on hypothetical genes is really a very traditional approach that becomes large-scale simply because of the parallel nature of the implementation. It resembles the successful approach used by the Europeans to achieve the complete sequence of the Saccharomyces cerevisiae genome (Goffeau et al. 1996). The results would significantly increase our functional knowledge of the genes within the microbial genomes thus far sequenced. Such annotation would be immediately applicable across orthologs and could dramatically improve the value of the sequenced genomes. This, in turn, would facilitate our ability to annotate new genomes as they appear. The proposal also reinforces the notion that the overwhelming value of bioinformatics is to generate hypotheses that can be tested experimentally. By enabling the community to join in this effort, we would also demonstrate that science really is the collaborative enterprise that requires all of our contributions, not just a select few. Finally, if this initiative succeeds, it would serve as a suitable model from which to begin the more daunting task of trying to annotate the functions of the complex eukaryotic genomes, such as the human genome.

##

**Box 1. HemK, a Very Highly Conserved Protein Methyltransferase**

During studies of the genetics of heme biosynthesis in E. coli, one gene, *hemK*, was found that had no immediately known protein product (Nakayashiki et al. 1995). Since one of the missing biosynthetic activities required for heme biosynthesis is protoporphyrinogen oxidase, the original authors suggested that the *hemK* gene might encode this enzyme. Subsequent *hemK* homologs were annotated as putative protoporphyrinogen oxidases and soon the “putative” was dropped. Then one group noticed that the gene product contained protein sequence motifs typical of DNA adenine methyltransferases. From then on, the annotations in GenBank alternated between these two assignments.

This *hemK* gene is of ubiquitous occurrence from humans to Chlamydia, and yet until 2002 its true biochemical function was unknown. At that point, two groups (Heurgue-Hamard et al. 2002; Nakahigashi et al. 2002) demonstrated that neither previous assignment was correct. Instead, they found that in E. coli the HemK gene product was an N5 glutamine methyltransferase that transferred a methyl group from S-adenosylmethionine to the amide nitrogen of a specific glutamine residue in the protein chain release factors *prfA* and *prfB*. Particularly noteworthy was the observation that the *hemK* gene is positioned immediately adjacent to the *prfA* gene in many microbial genomes! Here is a case where bioinformatics suggested strongly that the *hemK* gene encoded a methyltransferase, but an experiment was needed to identify the substrate. Since the adjacent gene in the genome encoded the substrate, it might have been possible to make that prediction too. There are additional paralogs of *hemK* in several genomes, but their biochemical activity and substrates remain to be identified.
